# GATA2 Deficiency and Epstein–Barr Virus Disease

**DOI:** 10.3389/fimmu.2017.01869

**Published:** 2017-12-22

**Authors:** Jeffrey I. Cohen

**Affiliations:** ^1^Medical Virology Section, Laboratory of Infectious Diseases, National Institute of Allergy and Infectious Diseases, National Institutes of Health, Bethesda, MD, United States

**Keywords:** GATA2, Epstein–Barr, chronic active Epstein–Barr virus, infectious mononucleosis, hydroa vacciniforme, smooth muscle tumors

## Abstract

GATA2 is a transcription factor that binds to the promoter of hematopoietic genes. Mutations in one copy of the gene are associated with haploinsufficiency and reduced levels of protein. This results in reduced numbers of several cell types important for immune surveillance including dendritic cells, monocytes, CD4, and NK cells, as well as impaired NK cell function. Recently, GATA2 has been associated with several different presentations of severe Epstein–Barr virus (EBV) disease including primary infection requiring repeated hospitalizations, chronic active EBV disease, EBV-associated hydroa vacciniforme with hemophagocytosis, and EBV-positive smooth muscle tumors. EBV was found predominantly in B cells in each of the cases in which it was studied, unlike most cases of chronic active EBV disease in which the virus is usually present in T or NK cells. The variety of EBV-associated diseases seen in patients with GATA2 deficiency suggest that additional forms of severe EBV disease may be found in patients with GATA2 deficiency in the future.

## The GATA Family of Transcription Factors

The GATA family of transcription factors consist of six proteins (GATA1 to GATA6) that contain two zinc finger-binding domains that bind to GATA sites on DNA (1, 2). GATA1 and GATA2 are important for hematopoiesis, with GATA1 important for development of red blood cells and platelets, and GATA2 for development of hematopoietic stem cells and progenitor cells. GATA3 is important for development of T cells. In contrast, GATA4 to GATA6 have critical roles in cardiac embryogenesis.

The GATA2 gene contains seven exons, five of which are translated. In addition to two zinc finger domains, the protein contains two transcriptional activation domains, a negative regulatory domain, and a nuclear localization signal (3). The protein undergoes a number of posttranslational modifications including phosphorylation, ubiquitination, SUMOylation, and acetylation (3). Mice that are homozygous knockouts for GATA2 die *in utero* due to a failure of hematopoiesis (4); in contrast, mice that are heterozygous for GATA-2 deficiency have reduced numbers of hematopoietic progenitor cells (5).

GATA2 is necessary for survival and renewal of hematopoietic stem cells and interacts with multiple transcription factors that regulate gene expression in hematopoietic stem cells. The quantity of GATA2 is critical for its activity, thus, reduced levels due to haploinsufficiency can have a profound phenotype. GATA2^+/-^ mice have fewer functional hematopoietic stem cells and granulocyte–macrophage progenitors in the bone marrow and the cells are impaired for self-renewal (6, 7).

## GATA2 Deficiency Phenotype and Mutations

GATA2 deficiency in humans, due to haploinsufficiency, has been associated with a wide array of diseases (8–11). These include hematologic disorders such as myelodysplastic syndrome, acute myelogenous leukemia, chronic myelomonocytic leukemia, aplastic anemia, as well as low numbers of monocytes, B cells, NK cells, dendritic cells, and neutrophils. Infectious complications include viral, bacterial, and fungal infections. Virus infections include human papillomavirus virus (HPV) infection that can transition to HPV-positive squamous cell carcinoma, or severe molluscum contagiosum, herpes simplex virus, varicella-zoster virus, cytomegalovirus, or Epstein–Barr virus (EBV) infection. Severe nontuberculous mycobacteria infections are commonly seen with GATA2 deficiency, while fungal infections include invasive aspergillosis, disseminated histoplasmosis, and recurrent candidiasis. Other complications reported in patients with GATA2 deficiency include pulmonary alveolar proteinosis, congenital lymphedema, panniculitis, erythema nodosum, venous thromboses, and deafness.

**Figure 1:**
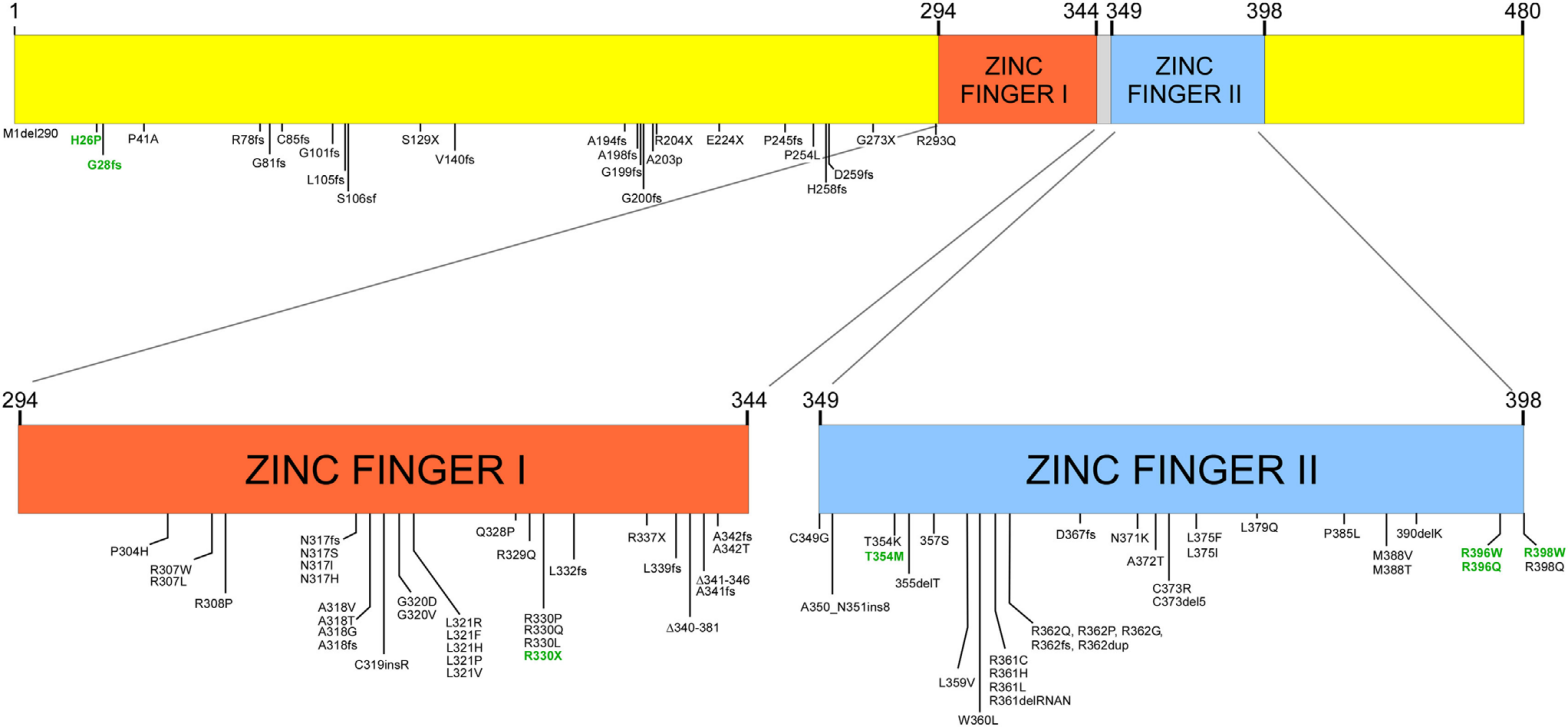
Structure of GATA2 and mutations described with GATA2 insufficiency and in patients with severe Epstein–Barr virus (EBV) disease [adapted from data in Ref. (8)]. Numbers in green represent mutations associated with severe EBV disease.

Many mutations have been detected in GATA (Figure 1), most of which are germ line, while somatic mutations have been reported in patients with leukemia (10). While most mutations have been reported in the coding region of the gene, mutations in regulatory regions such as in the enhancer region of intron 5 and the 5′ leader sequence result in reduced transcription (12). Mutations associated with disease are most often in one of the two zinc finger-binding domains; these include amino acid substitutions, frameshift mutations, and insertions and deletions. These result in either protein dysfunction or reduced transcription from one of the two alleles. Thus, deletion of one allele, mutations in non-coding regulatory regions of the gene, or mutations in one allele can result in haploinsufficiency due to reduced transcription, loss of protein expression, or expression of a non-functional protein. Most cases are due to *de novo* mutations while about one-third are inherited as an autosomal dominant condition. In some cases, mutations have not been identified, but transcription of only one of the two alleles has been demonstrated.

## Hematologic Findings in GATA2 Deficiency Important for Control of Virus Infections

**Figure 2:**
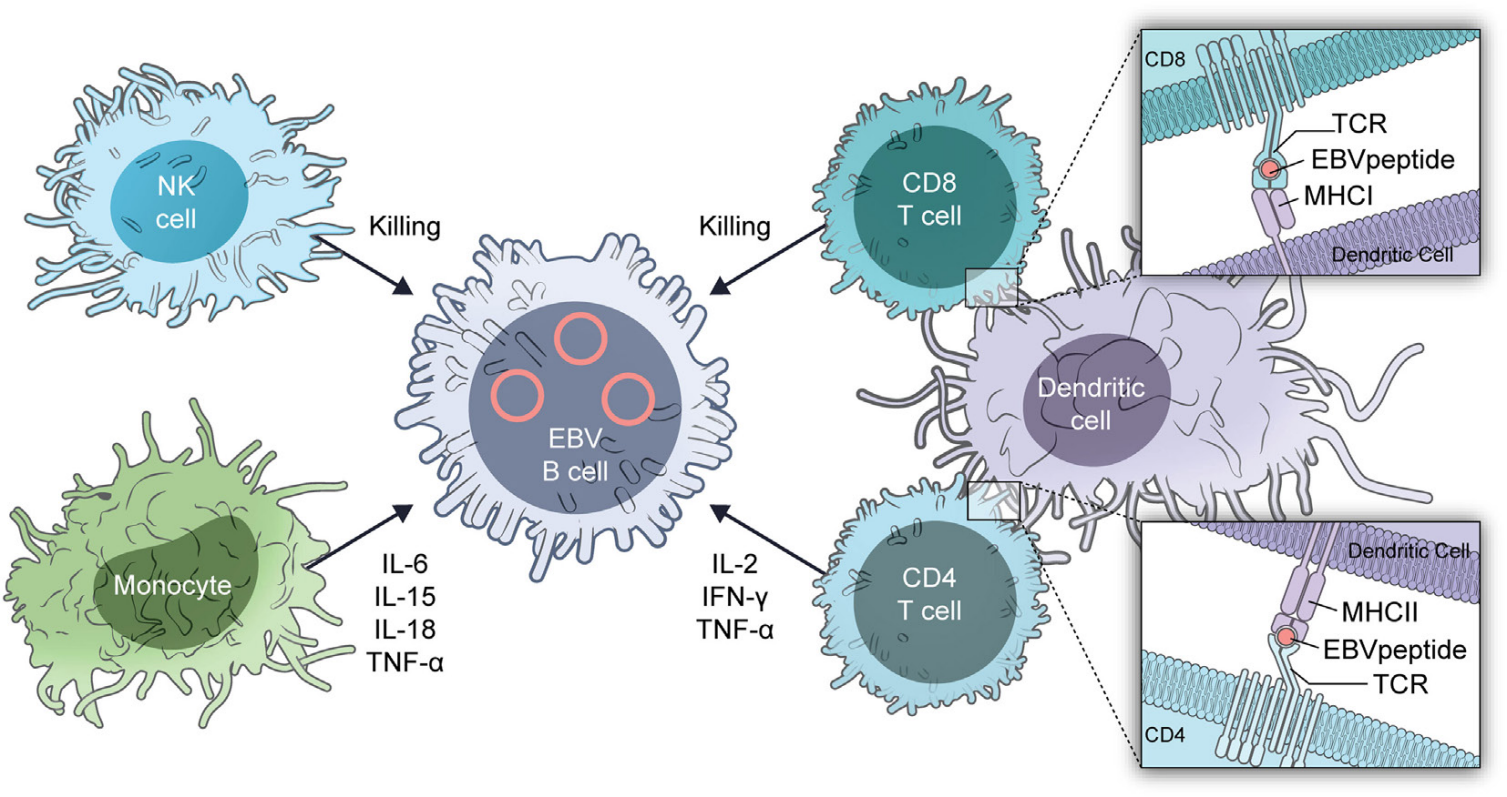
Immune system cells important for controlling Epstein–Barr virus (EBV)-infected B cells.

The mechanism for the predilection of patients to severe EBV infections is almost certainly multifactorial (Figure 2). Absence or reduction in the numbers of dendritic cells with GATA2 insufficiency (13) can reduce recognition of EBV by the immune system. Dendritic cells are critical for presentation of EBV antigens to T cells (14) and EBV in turn inhibits dendritic cell maturation (15).

Reduction in the number of monocytes in patients with GATA deficiency (11, 16) reduces cytokine responses with reduced IFN-γ and IL-12. EBV inhibits MHC class I, class II, ICAM1, CD80, and CD86 expression on monocytes, which inhibit T cell proliferation and antigen presentation by the cells (17, 18). Patients with GATA2 deficiency have a decrease in the number of hematogones (precursor B cells) and B cells including naïve B cells, with a relative increase in the number of memory B cells (19, 20). B cells are important for antigen presentation and help to activate T cells.

GATA2 deficiency is associated with a reduction in the number of NK cells and reduced NK cell-mediated cytotoxicity (11, 16, 21). In addition, there is a reduction in the number of immature (CD56^bright^) NK cells (21), while other NK cell markers indicative of maturity, such as killer cell immunoglobulin-like receptors, are increased (14). NK cells are critical for control of primary infection with EBV (22) as well as to inhibit transformation of B cells by the virus (23).

Like NK cells, CD8 T cells in patients with GATA2 deficiency are skewed toward a more mature, terminally differentiated phenotype (16). The reduction in naïve T cells may also impair control of EBV. These patients also have a reduction in the number of CD4 cells with an inverted CD4/CD8 ratio. Both CD4 and CD8 cells are important for control of EBV infection. During primary infection, there is a massive expansion of CD8 cells and these cells recognize both lytic and latent viral antigens (14). CD4 cells are important for control of primary infection, but there is less proliferation of CD4 than CD8 cells. During convalescence, up to 5% of CD8 cells are directed against EBV antigens, while less than 1% of CD4 cells recognize EBV proteins.

GATA2 may also affect herpesvirus latency. GATA2 controls the expression of at least two latency associated genes in human cytomegalovirus, UL144 and LUNA (24). GATA2 also increases expression of cellular IL-10 (25), which is important for CMV latency (26). At least two EBV latency associated genes, EBV-encoded RNA and latent membrane protein 1 (LMP1), upregulate cellular IL-10. Both CMV (27) and EBV (28) encode IL-10 homologs, which are important for immune evasion.

Studies of gene transcription in EBV-transformed B cells from patients with GATA2 deficiency due to haploinsufficiency compared with controls showed significant differences in expression of 102 genes (12). Several genes known to have an important role in EBV transformation, including NOTCH1, TRAF2, and TRAF3, were downregulated in EBV-transformed B cells derived from GATA2-deficient patients compared with controls. EBNA2, which is essential for EBV latency, is a functional homolog of a constitutive NOTCH receptor and LMP2, which also has a critical role in EBV latency, activates NOTCH to increase survival of B cells. Therefore, reduced expression of NOTCH1 in cells from patients with GATA2 deficiency may reduce the ability of the virus to maintain latency. LMP1, which is essential for EBV latency, interacts with TRAF2 and 3 to activate the NF-κB pathway and maintain latency. Thus, impaired expression of NOTCH1, TRAF2, and TRAF3 in cells from patients with GATA2 deficiency may reduce the ability of the virus to maintain latency. This could result in increased virus replication resulting in a higher viral load, more severe primary infection (infectious mononucleosis), and increased infection of additional cells resulting in more severe EBV disease.

## EBV Disease Associated with GATA2 Deficiency

Patients with GATA2 deficiency may present with a variety of EBV-associated diseases. These patients may have severe complications with EBV infectious mononucleosis. Identical twins with GATA2 insufficiency, due to a stop codon (R330X) in one allele, presented with symptoms of infectious mononucleosis; one had three hospitalizations for anorexia and dehydration accompanied by anemia, fatigue, weight loss, and fever for 3 months with 20,600 copies of EBV DNA/ml of blood (29). Her course was complicated by numerous infections including *Neisseria meningitditis* bacteremia and Salmonella enteritis. Her sister also presented with EBV infectious mononucleosis complicated by fatigue, weight loss, anemia, thrombocytopenia, and hypotension with 8,900 copies of EBV DNA/ml of blood. Both sisters had reduced numbers of monocytes and CD4, CD8, B, and NK cells. They had a persistently elevated EBV load in the blood after their symptoms of primary infection resolved. Both had a history of herpes stomatitis and severe warts and both did well after hematopoietic stem cell transplant. A third patient with a missense mutation (R396Q) in one allele of GATA2 presented with EBV infectious mononucleosis and was hospitalized twice, once for severe fatigue with headache and rash, and a second time for dehydration and malaise with 44,000 copies of EBV DNA/ml of blood. She had a history of herpes stomatitis and *Staphylococcus aureus* cellulitis and reduced numbers of monocytes and CD4, CD8, B, and NK cells. Both the bone marrow and a lymph node contained EBV-positive lymphocytes. She also had persistently elevated EBV DNA in the blood after resolution of her infectious mononucleosis symptoms.

A 29-year-old woman with GATA2 insufficiency with null allelic loss of one copy of the gene and a positive EBV IgM viral capsid antibody in the serum and cerebrospinal fluid, indicative of acute infection, developed a demyelinating polyradiculopathy (30). EBV DNA was found in the cerebrospinal fluid, but no EBV DNA was detected in a sural nerve biopsy. She had a history of recurrent pneumonia, severe varicella, and severe genital warts with cervical dysplasia. Her neurological disease responded to intravenous immunoglobulin and corticosteroid therapy.

A 22-year-old man with GATA2 insufficiency due to a missense mutation (T354M) in one allele of GATA2 presented with chronic active EBV disease (29). This is a very rare disorder characterized by infiltration of tissues with EBV-positive lymphocytes, a high level of EBV in the blood, and persistent or intermittent symptoms lasting 6 months or more. He had evidence for a primary EBV infection and persistent splenomegaly and pancytopenia and both the spleen and an adjacent lymph node showed EBV-positive B cells. He had 2,770 copies of EBV DNA/ml of blood. He had reduced numbers of monocytes and CD4, B, and NK cells, but normal numbers of CD8 cells. The patient also had *Mycobacterium abscessus* and died from his mycobacterial infection.

A 24-year-old woman who expressed only one allele of GATA2, but had no definite mutation in the gene, presented with EBV hydroa vacciniforme (29, 31). This EBV disorder is characterized by a vesicular rash and infiltration of the skin with EBV-infected lymphocytes in response to sun exposure; it may progress to a systemic disease with EBV-positive T or NK cell lymphoma. The patient had large skin lesions that were EBV-positive as well as EBV in the lung, intestine, skeletal muscle, and cerebrospinal fluid. There were 6.4 million copies of EBV DNA/ml of blood. Her course was complicated by multiple infections including *Mycobacterium avium* complex, histoplasmosis, and enterococcus bacteremia. She had reduced numbers of monocytes and CD4, B, and NK cells, but normal numbers of CD8 cells. She developed an EBV-positive T cell lymphoma of the lung, gastrointestinal tract, and skin as well as hemophagocytic lymphohistiocytosis. She did well after hematopoietic stem cell transplant.

Two patients with GATA2 insufficiency have been reported with EBV-positive smooth muscle tumors (29, 32, 33). One had a missense mutation (R398W) in GATA2 and EBV-positive leiomyosarcomas involving the posterior orbit, liver, colon, and uterus. She had a history of disseminated *M. avium* involving the skin, blood, and intestine, herpes simplex esophagitis, warts, and chronic myelomonocytic leukemia. She had reduced numbers of monocytes and CD4, B, and NK cells, but normal numbers of CD8 cells. She underwent hematopoietic stem cell transplant for the leukemia, but died of a respiratory tract infection. The second patient also had a missense in GATA2 (R396W) and an EBV-positive spindle cell tumor involving the liver with 3,350 copies of EBV DNA/ml of blood. A positron emission tomographic scan showed multiple metabolically active lesions in the liver, spleen, mediastinum, hilum, scapula, vertebrae, and pelvis. He had a history of recurrent pneumonia, *Mycobacterium szulgai* pneumonia, and *M. avium* in a bronchoalveolar lavage. He had reduced numbers of monocytes and CD4, CD8, B, and NK cells. He underwent hematopoietic stem cell transplant and his immunologic abnormalities resolved and his lesions resolved or remained stable.

A patient with a frameshift (G28fs) and missense mutation (H26P) in exon 2 of GATA2 had myelodysplastic syndrome, progressive pancytopenia, and an EBV-positive T-cell non-Hodgkin lymphoma of the nasopharynx (34). He died despite therapy with corticosteroids and rituximab.

## Studies of EBV Disease in Patients with GATA2 Deficiency

Analysis of 51 patients with GATA2 deficiency showed that the median level of EBV DNA in the blood of EBV seropositive patients without EBV disease was 117 copies/ml, while the level was 14,750 copies/ml in persons with EBV disease (29). An additional patient has been reported with GATA2 deficiency, diffuse parenchymal lung disease, acute EBV infection and persistent viremia, but no other EBV complications (35). Patients with GATA2 deficiency and severe EBV disease have different patterns of EBV latency gene expression in their peripheral blood and some have expression of EBV BZLF1 indicating that the virus is undergoing lytic gene expression. Patients with severe EBV disease and GATA2 deficiency have high plasma levels of IP-10 (an interferon response gene) and TNF-α, and low levels of IL-1-β compared with normal controls. IP-10 and TNF-α are Th1 cytokines important for cellular immunity.

## Treatment for EBV Disease Associated with GATA2 Deficiency

Definitive treatment for GATA2 deficiency requires hematopoietic stem cell transplant (36). In the largest series to date, 14 patients underwent non-myeloablative allogeneic hematopoietic stem cell transplant (31). Eight of the 14 were alive a median of 3.5 years later with reconstitution of their immune system. Survivors received peripheral blood stem cells from matched related, unrelated, or haploidentical related donors, or umbilical cord blood. The latter group had the lowest survival rate. Deaths were often due to sepsis, graft-versus-host disease, acute myelogenous leukemia, or acute respiratory distress syndrome. Transplantation can be especially challenging in this disease due to concurrent infections and preexisting leukemia or transformation of myelodysplastic syndrome into leukemia. Cases of relapsed disease after hematopoietic stem cell transplant and graft rejection suggests that myeloablative conditioning may be preferred, although preexisting comorbidities including severe infections may make myeloablation challenging in these patients.

Antiviral therapy is generally ineffective for EBV disease associated with GATA2 deficiency. EBV malignancies are due to proliferation of latently infected lymphocytes or epithelial cells. The viral DNA replicates in these cells using the host cell polymerase, which is insensitive to antivirals; in contrast, lytic virus replication in epithelial cells, which occurs during virus shedding in healthy persons or in those with oral hairy leukoplakia is sensitive to antivirals.

Treatment of some patients with GATA2 insufficiency with IFN-α resulted in increased numbers of NK cells and/or function of the cells, but did not increase the number of CD56^bright^ cells (21). Rituximab may have a role for EBV-positive B cell tumors, but in the absence of reconstitution of the immune system, the disease can recur with CD20-negative B cell tumors (37). Treatment with third party EBV-specific T cells might provide temporizing therapy prior to hematopoietic stem cell transplant (38).

## Future Directions

GATA2 deficiency has been associated with a large number of diseases particularly hematologic and infectious diseases. At present, it is unclear why certain patients present with specific complications associated with GATA2 deficiency while others do not. While severe viral infections are significantly more common in persons with GATA2 null mutations (11), these infections were also associated with missense and regulatory mutations. However, within large families, different family members with the same mutation can have very different presentations (34). In our patients with severe EBV disease, a variety of mutations in GATA2 including missense mutations, stop codons, null allelic loss of one copy, and unialleleic gene expression were all observed. Some patients have high levels of EBV in the blood and complications of EBV, while others have levels of EBV that are seen in healthy controls. This does not appear to be specific to particular mutations in GATA2, but likely reflects the effects of polymorphisms, mutations, or epigenetic changes in other genes that might modify the phenotype of patients with GATA2 deficiency. This phenomenon of modifier genes has been well described in other genetic disorders (39) and with increasing resources and expertise in computation and bioinformatics, the role of modifier genes affecting GATA2 function may help to explain the different EBV phenotypes observed with GATA2 deficiency. In addition, environmental differences, the immune status of the patient, or coinfections at the time of primary EBV infection might also affect whether some patients with GATA2 deficiency develop severe EBV disease.

GATA2 deficiency is a relatively newly described disorder, and the wide array of severe EBV diseases observed in patients with this disorder suggest that additional presentations of EBV disease may be associated with GATA2 deficiency in the future. In addition, polymorphisms in the GATA2 gene or its regulatory elements might be associated with less severe presentations of EBV disease. Thus, additional studies of GATA2 should provide insights into the role of this gene in control of EBV and other infectious diseases.

## Author Contributions

The author confirms being the sole contributor of this work and approved it for publication.

## Conflict of Interest Statement

The author declares that the research was conducted in the absence of any commercial or financial relationships that could be construed as a potential conflict of interest.
